# Weekly phototherapy is an effective therapy for patients with vitiligo

**DOI:** 10.3892/mi.2023.118

**Published:** 2023-10-30

**Authors:** Jing-Yu Wang, Shu-Lan Yao, Xin-Yi Hou, Hai-Lu Xiao, Bin Lu

**Affiliations:** 1 School of Clinical Medicine, Jining Medical College, Rencheng Campus, Jining, Shandong 272113, P.R. China; 2 Department of Dermatology, Affiliated Hospital of Jining Medical College, Jining, Shandong 272113, P.R. China

**Keywords:** vitiligo, 308 nm excimer light, number of phototherapy sessions, treatment, skin

## Abstract

The current strategies for the treatment of vitiligo using phototherapy usually involve treatment for two-three times per week; however, in practice, the number of patient sessions does not meet this standard. The present study found that phototherapy once a week was also effective. The present study was designed to examine the efficacy of weekly light therapy. For this purpose, 296 patients with vitiligo were included and divided into five sub-samples of the neck, face, trunk, extremities and scalp according to the site of phototherapy, and were treated once or twice weekly with phototherapy. The difference in efficacy between phototherapy performed once and twice weekly was observed using a Chi-squared test. It was concluded that there was a minimal difference between phototherapy performed twice weekly compared to once weekly for the treatment of vitiligo on the face, neck, torso, limbs and scalp. Thus, phototherapy once a week is valid for the treatment of vitiligo, although weekly light therapy takes longer to restore color for the first time.

## Introduction

Vitiligo is an acquired autoimmune disease involving the loss of melanocytes as the main characteristic feature, resulting in colorless patches (1,2). It affects 0.5-2% of the population among adults and children worldwide (2). Although some guidelines for the treatment of vitiligo have been released (3,4), the treatment of vitiligo remains a difficult challenge in dermatology. There are various treatments available, such as surgery, topical and systemic immunosuppressants, make-up and phototherapy (3-6).

Phototherapy, the main method used for the treatment of vitiligo, has various efficacies. Among these, narrow-band ultraviolet B (NB-UVB) and ultraviolet A (UVA) have been used for decades with good efficacy. NB-UVB is one of the main treatments used for vitiligo. Due to its efficacy and minimal side-effects, it has become the mainstay for the widespread treatment of vitiligo (7). The Vitiligo Working Group (VWG) recommended that the use of NB-UVB two or three times per week is an optimal weekly frequency for the treatment of vitiligo) (7).

Monochromatic excimer light (MEL, 308 nm) was developed for use in dermatology (8), and has become the mainstay treatment for vitiligo due to its good efficacy and limited side-effects. The specification of the UV light therapy instrument used in this experiment is: Dema XECL-308. Despite its widespread use, there are almost no standardized guidelines available on the dose and frequency of MEL application for the treatment of vitiligo.

Therefore, the present study aimed to examine the different efficacy of the use of different frequencies of MEL administration in a larger number of patients with vitiligo.

## Patients and methods

### Study approval and design

The research program was confirmed by the Medical Ethics Committee of the Affiliated Hospital of Jining Medical University (Jining, China; ethical approval no. 2022C261). The study was performed at the Dermatology Department of the Affiliated Hospital of Jining Medical University. Informed consent was obtained from all the patients and/or their guardians.

A total of 296 subjects were divided into five sub-samples according to the different areas of phototherapy: Neck, face, trunk, extremities and scalp. The present study was designed as a cross-sectional study. The median age of the patients was 15 years, with a maximum age of 70 years and a minimum age of 2.2 years. The patients were then subdivided into five sub-samples by site. A Chi-squared test, rank sum test and a two independent samples t-test were used to confirm whether the frequency of phototherapy yielded a difference in the frequency of the treatment or the number of initial color restorations. There was no difference in the former, leading to conclusion 1: It cannot yet be assumed that there is a difference in the treatment efficacy between phototherapy performed once or twice-weekly, and that phototherapy performed once weekly is effective; there was no difference in the latter, leading to conclusion 2: It cannot yet be assumed that there is a difference between phototherapy performed weekly and twice weekly in terms of the number of initial color restorations, so that with the same number of color restorations, weekly phototherapy requires a longer treatment duration than phototherapy performed twice weekly.

### Patients

Patients were recruited from the outpatient clinic of the Department of Dermatology at the Affiliated Hospital of Jining Medical College for a period of 2 years and 3 months, i.e., between June, 2018 and September, 2020. Patients with non-segmental vitiligo were included in the present study. Adult patients with symmetrical vitiligo with lesions of at least 10 cm^2^ and at least 3 months of age were included in the study. The exclusion criteria were topical or systemic treatment 4 weeks prior to the study, phototherapy 12 weeks prior to the study, previous skin cancer or radiotherapy at the treatment site, pregnancy and other contraindications to phototherapy (9).

### Statistical analysis

In the present study, IBM SPSS Statistics 26 software was used to analyze the data. Data were analyzed using a Chi-squared test, as well as a one-sample rank sum test and a two independent samples t-test. A value of P<0.05 was considered to indicate a statistically significant difference.

## Results

Between June, 2018 and September, 2020, a total of 296 patients with a clinical diagnosis of non-segmental vitiligo were included in the present study. The characteristics of the patients including sex, age, medical history (duration of vitiligo), number of treatments until repigmentation was achieved, the dose at which color was restored, the area of the body and effects under different treatment frequencies are presented in Table I.

In Table II, ‘healing’ indicates that the skin lesions of patients with vitiligo had completely returned to normal following treatment; ‘effective’ indicates that the skin lesions of patients with vitiligo notably improved following treatment, but had not recovered completely; ‘invalid’ indicates that the skin lesions of patients with vitiligo remained almost unaltered following treatment; and ‘total’ represents the total number of patients with vitiligo. In Table II, the Chi-squared test was used to determine difference in efficacy between phototherapy performed once and twice weekly. As shown by the results, that the difference in the efficacy of the frequency of phototherapy in the five regions of the face, neck, trunk, extremities and scalp was not statistically significant. Thus, it cannot yet be assumed that there is a difference in the therapeutic efficacy between phototherapy performed once or twice weekly.

The present study then examined whether there was a difference in the number of treatments to achieve recoloration with different phototherapy frequencies. As shown in Table III, the one-sample rank sum test was used for the male, female, face, neck, trunk and scalp samples as they did not meet normality, and two independent samples t-tests were used for extremity samples which met normality, all with P-values >0.05, indicating no statistically significant differences. Thus, it cannot yet be assumed that there is a difference in the number of phototherapy sessions performed once vs. twice weekly as regards the restoration of color. With the same number of times, phototherapy performed once weekly requires a longer treatment duration than phototherapy performed twice weekly.

## Discussion

Research has indicated that following 308 nm excimer laser treatment, the number and volume of melanocytes in vitiligo lesions are increased compared to previous ones, and the migration, activation and production of melanin induces pigmentation on the skin surface (10). Compared to NB-UVB phototherapy (311-313 nm), 308 nm excimer laser appears to augment and expedite the repigmentation process (11,12). In addition, the 308 nm excimer laser enhances and accelerates the pigmentation process to a greater extent than NB-UVB phototherapy (13), which not only provides more rapid results and shorter treatment durations, but also limits the accumulation of UV radiation, and may thus reduce the risk of developing skin cancer (14-17). The 308 nm excimer light is currently replacing NB-UVB as a more effective treatment for vitiligo. It has been reported that there is no necessary link between the course of vitiligo and disease progression and treatment outcomes (18). Therefore, the present study did not consider the duration of the disease and treatment progression, but used a cross-sectional approach, focusing on the frequency of treatment. The current optimal treatment regimen for vitiligo is three weekly phototherapy sessions (7); however, it has been found that a once weekly session of phototherapy is also effective. The Consensus on the Treatment of Vitiligo (2021 Edition) also recognizes the effectiveness of weekly phototherapy (19), and the optimal frequency of excimer light therapy remains to be investigated. It has been demonstrated that the final repigmentation results of the 308-nm excimer laser are not associated with the frequency of treatment (20). This provides strong evidence to support the findings of the present study. In addition, numerous patients in remote areas are unable to complete phototherapy twice or thrice weekly, and it is thus critical to explore the effectiveness of weekly phototherapy. Therefore, the present study examined whether there was any difference between light therapy performed once and twice weekly in terms of treatment efficacy and the number of initial color restorations to determine whether light therapy performed twice weekly is more effective. The results revealed that there was no significant difference between phototherapy performed once or twice weekly in terms of treatment efficacy and the number of initial color restorations. Weekly light therapy was effective; however, for the same number of treatments, weekly light therapy took longer than light therapy performed twice weekly to restore color for the first time.

The study population examined herein was selected from patients who visited the outpatient clinic during a total of 2 years and 3 months from June, 2018 to September, 2020. The limitations of the present study are the use of cross-sectional surveys, too wide an age span and the lack of follow-up, which may lead to survey bias. A larger sample data and a homogeneous population need to be selected in the future to validate the results obtained herein. In addition, the number of patients with vitiligo in the neck and scalp areas was minimal in the study sample and the results may thus be subject to error.

In conclusion, the present study demonstrated that the difference in efficacy between phototherapy performed twice or once weekly was not significant. Although weekly light therapy is effective, it requires a longer duration to be effective compared with light therapy performed twice weekly.

## Figures and Tables

**Table I:** Basic information of the patients with vitiligo treated with phototherapy once vs. twice a week.

No. of light treatments	Sex (no. of patients)	Age (years)	Medical history (duration of vitiligo; months)	No. of treatments until repigmentation was achieved	Number of doses required to achieve recoloration	Affected body part (no. of patients)	Effects of treatment (no. of patients)
Once a week	Male (79)	18.69±12.24	9.00±13.02	4.94±3.51	7.31±6.87	Face (66)	Healing (45)
	Female (84)					Neck (17)	Effective (117)
						Torso (44)	Invalid (1)
						Extremities (28)	
						Scalp (8)	
Twice a week	Male (73)	20.71±14.36	6.55±10.08	5.15±3.57	7.82±7.95	Face (55)	Healing (33)
	Female (60)					Neck (16)	Effective (98)
						Torso (25)	Invalid (2)
						Extremities (27)	
						Scalp (10)	

Data for age, medical history, no. of treatments until repigmentation and recoloring doses are presented as the mean ± SD.

**Table II:** Comparison of the efficacy of different phototherapy sessions for patients with vitiligo treated with phototherapy in the present study.

Affected body part	No. of light treatments per week	Healing (no. of patients)	Effective (no. of patients)	Invalid (no. of patients)	Total (no. of patients)	P-value
Face	1	25	41	0	66	0.354
	2	19	36	0	55	
Neck	1	3	13	1	17	0.971
	2	3	13	0	16	
Torso	1	10	34	0	44	0.070
	2	5	20	0	25	
Extremities	1	5	23	0	28	2.185
	2	4	21	2	27	
Scalp	1	2	6	0	8	0.064
	2	2	8	0	10	
Total		78	215	3	296	

**Table III:** Difference in the number of treatments when reaching recoloration for patients with vitiligo treated with different frequencies of phototherapy.

	Male		Female		Face		Neck		Trunk		Extremities		Scalp	
Treatment times per week	1	2	1	2	1	2	1	2	1	2	1	2	1	2
No. of treatments until repigmentation was achieved (mean ± SD)	4.99±3.24	5.49±3.259	4.90±3.76	4.73±3.912	3.68±1.824	3.80±1.976	4.47±2.095	5.19±3.124	4.05±2.134	4.88±3.887	9.11±5.343	8.16±4.661	6.75±3.454	5.60±3.134
	5.23±3.248		4.83±3.811		3.74±1.888		4.82±2.627		4.35±2.894		8.66±5.007		6.11±3.234	
P-value	0.217		0.600		0.802		0.709		0.445		0.318		0.360	

## Data Availability

The datasets used and/or analyzed during the current study are available from the corresponding author on reasonable request.
